# Revealing the pharmacological effects of Remodelin against osteosarcoma based on network pharmacology, acRIP-seq and experimental validation

**DOI:** 10.1038/s41598-024-54197-4

**Published:** 2024-02-13

**Authors:** Jia Gao, Peili Xu, Feng Wang, Wenjie Zhang, Meipeng Min, Rafi Urba, Lei Fan

**Affiliations:** 1 Department of Orthopedics, The Second Affiliated Hospital of Nanjing Medical University, Nanjing, 210011 Jiangsu People’s Republic of China; 2 Department of Orthopedics, Sir Run Run Hospital, Nanjing Medical University, Nanjing, 210011 Jiangsu People’s Republic of China

**Keywords:** Bone cancer, Pharmacology

## Abstract

Osteosarcoma (OS) is the most common primary malignant tumor of bone. Remodelin, an inhibitor of the N (4)-Acetylcytidine (ac4C) acetylation modifying enzyme N-acetyltransferase 10 (NAT10), has been shown to have therapeutic effects on cancer in several studies, and our previous studies have confirmed the inhibitory effect of Remodelin on OS cells, however, the mechanism of action has not yet been elucidated. We used network pharmacological analysis to quantify the therapeutic targets of Remodelin against OS. acRIP-seq and RNA-seq were performed to investigate the inhibitory activity of Remodelin on acetylation and its effect on the transcriptome after intervening in OS cells U2OS with Remodelin in vitro. Key target genes were deduced based on their pharmacological properties, combined with network pharmacology results and sequencing results. Finally, the deduced target genes were validated with vitro experiments. Network pharmacological analysis showed that 2291 OS-related target genes and 369 Remodelin-related target genes were obtained, and 116 overlapping genes were identified as Remodelin targets for OS treatment. Sequencing results showed that a total of 13,736 statistically significant ac4C modification peaks were detected by acRIP-seq, including 6938 hypoacetylation modifications and 6798 hyperacetylation modifications. A total of 2350 statistically significant mRNAs were detected by RNA-seq, of which 830 were up-regulated and 1520 were down-regulated. Association analyses identified a total of 382 genes that were Hypoacetylated-down, consistent with inhibition of mRNA acetylation and expression by Remodelin. Five genes, CASP3, ESR2, FGFR2, IGF1 and MAPK1, were identified as key therapeutic targets of Remodelin against OS. Finally, in vitro experiments, CCK-8 and qRT-PCR demonstrated that Remodelin indeed inhibited the proliferation of OS cells and reduced the expression of three genes: ESR2, IGF1, and MAPK1. In conclusion, ESR2, IGF1 and MAPK1 were identified as key therapeutic targets of Remodelin against OS. This reveals the target of Remodelin's pharmacological action on OS and provides new ideas for the treatment of OS.

## Introduction

OS stands as the prevailing primary malignant tumor of bone, exhibiting the highest prevalence among adolescents and young adults during the pubertal growth spurts^1^. Chemotherapy and surgery are the conventional methods of treating OS, however, these treatments can cause long-term toxic effects and have a detrimental effect on one's quality of life. Enhanced molecular analysis has unveiled distinct categories of OS, facilitating accurate management of osteosarcoma while mitigating the adverse effects of chemotherapeutic agents or even attaining superior therapeutic outcomes. These molecular targets have the potential to lay the groundwork for the advancement of novel treatments for this tumor.

The epitranscriptome, which consists of a variety of post-transcriptional chemical changes to RNA, plays a crucial role in controlling gene expression. Increasingly, it appears that RNA modifications may be a viable option for cancer treatment^2^. Ac4C is an emerging form of epitranscriptome that improves transcript stability and translational efficiency^3^. Furthermore, studies have demonstrated that ac4C alteration is implicated in a variety of biological activities, such as osteogenesis, AIDS, myocardial infarction, and cancer^2,4–9^. However, previous research on cancer in this area has been limited to colon, stomach, bladder, and multiple myeloma cancers^2,5,8,9^. And there have been no studies on OS.

NAT10, the sole protein encompassing both an N-acetyltransferase structural domain and a nucleotide-binding region, is regarded as an ac4C ‘writer’ protein responsible for governing RNA modification mechanisms. In cancer, NAT10 is usually expressed at high levels to maintain mRNA acetylation, which in turn promotes mRNA stability and translation^10^. Remodelin, as a novel small molecule, has been shown to reduce NAT10 activity^11^, thus preventing mRNA acetylation, and has been shown to be an effective treatment for various types of tumors^2^. In our previous study^12^, it was found that Remodelin can inhibit the growth of OS by suppressing the expression of NAT10 in osteosarcoma cells, but its specific mechanism of action remains to be elucidated.

To further investigate the mechanism of action of Remodelin on OS, we used a network pharmacology approach to search for therapeutic targets of Remodelin against OS. AcRIP-seq and RNA-seq were performed after in vitro intervention with Remodelin in OS cells U2OS to explore its effects on ac4C modification and the transcriptome. Finally, genes were deduced from its pharmacological reduction of acetylation and mRNA expression, combined with network pharmacology results and sequencing results. The network pharmacology results showed that 116 overlapping genes were identified as targets of Remodelin for the treatment of OS, a total of 382 genes that were Hypoacetylated-down and differed more than twofold were identified after association analyses by acRIP-seq and RNA-seq, Consistent with the inhibition of mRNA acetylation and expression by Remodelin. The combined analysis of network pharmacology results and sequencing results predicted that five genes, CASP3, ESR2, FGFR2, IGF1, and MAPK1, were the therapeutic targets of Remodelin for OS. Finally, with vivo experimental validation, ESR2, IGF1, and MAPK1 were identified as key therapeutic targets of Remodelin for OS. This reveals the target of Remodelin's pharmacological action on OS and provides new ideas for the treatment of OS.

## Materials and methods

### Network pharmacology analysis

#### Drug target of Remodelin

The molecular structure of Remodelin (sdf format) was obtained from the PubChem database (https://pubchem.ncbi.nlm.nih.gov/). Potential targets for Remodelin were screened from the following databases: the PharmMapper (http://lilab.ecust.edu.cn/pharmmapper/) and the Swiss Target Prediction (http://www.swisstargetprediction.ch/), In PharmMapper, input Remodelin's sdf format file for targets, where select target set selects Human Protein Targets Only (v2010, 2241), Convert uniprot to gene name. In Swiss Target Prediction, Remodelin's SMILE code is entered to predict the target. The targets obtained from both databases were merged to obtain Remodelin targets against OS.

#### Disease target of OS

Potential targets for OS were obtained by screening in the following databases: Online Mendelian Inheritance in Man (OMIM, https://omim.org/) and DisGeNET (https://disgenet.org/). In the OMIM database, Advanced Search selects Gene Map in which to search for OS-related disease targets. In the DisGeNET database, the Disease ID of OS was selected as C0029463 for the search of OS-related disease targets. Targets obtained from both databases were merged to obtain targets for OS. Venn diagrams were drawn to analyze overlapping genes and obtain potential targets of Remodelin for OS.

#### Construction of the protein–protein interaction (PPI) network

PPI analysis is a bioinformatics approach to analyze possible interactions between multiple proteins based on the String database, which can analyze the interaction strengths and weaknesses of a group of proteins, comprehensively examine the protein interaction network, and finally, screen obtain the key targets. Importing our obtained potential targets of Remodelin for OS into the String database for PPI networks, Organism chose Homo sapiens, meaning of network chose evidence, and the Minimum required interaction score was set to medium confidence (0.400). The results (tsv format) were imported into Cytoscape for visualization and analysis of complex relationships between genes.

### Sequence validation

#### Cell culture

The human OS cell lines U2OS and MNNG/HOS were purchased from China Typical Culture Preservation Center (Wuhan, China). U2OS cultured in RPMI 1640 medium (KeyGEN Biotech, China) supplemented with 10% fetal bovine serum (Gibco, USA). MNNG/HOS cultured in α-MEM medium (Gibco, USA) supplemented with 10% fetal bovine serum (Gibco, USA). Cells were stored in a humidified incubator containing 5% CO_2_ at 37 °C. Remodelin hydrobromide was purchased from (MCE, China).

#### RNA-seq analysis

TRIzol reagent was utilized to isolate total RNA from OS cells, both with and without Remodelin treatment. The Dynabeads mRNA Purification Kit (61006, Invitrogen) was used to purify Poly (A) + mRNA from total RNA, which was then sequenced on an Illumina NovaSeq 6000 platform by Epibiotek (Guangzhou, China). The human genome version 38 (GRCh38) was mapped with HISAT2 (v2.1.0) and then HT-seq (v0.7.2) was used to calculate the reads. Genes exhibiting a P value of less than 0.05 and a fold change of more than 1.2 were deemed to be differentially expressed.

#### AcRIP-seq analysis

Epibiotek (Guangzhou, China) was the primary source of support for AcRIP-seq and subsequent data analyses. TRIzol reagent was used to extract total RNA from OS cells treated with Remodelin or control OS cells, which was then digested with DNase I and fragmented into 100–200 nt oligonucleotides using RNA fragmentation reagents (ab252215, Ambion). Following the preservation of 50 ng of fragmented RNA as the input, the remaining (150 μg) was employed for RNA immunoprecipitation utilizing ac4C antibody (Abcam). The Ac4C RNAs were subjected to immunoprecipitation using Dynabeads Protein G (10004D, Invitrogen) and subsequently retrieved using HiPure cell miRNA (R4311-03, Magen, Guangzhou, China). The input RNA and ac4C-enriched RNA samples underwent extraction of ribosomal RNA. The EpiTM mini longRNA-seq kit (E1802, Epibiotek) was used to construct RNA sequencing libraries for input RNA (RNA-seq) and ac4C-enriched RNA (acRIP-seq) at the same time, followed by deep sequencing on the Illumina NovaSeq 6000 using the PE150 strategy with two separate biological replicates. The HISAT2 software (v2.1.0) was utilized to align the reads with the human genome GRCh38/hg38. The ExomePeak R package (v2.13.2) was then utilized to detect ac4C peaks. The ac4C peaks with a fold change of more than 2.0 and a p value of less than 0.05 were chosen.

#### Functional enrichment analysis

Gene ontology (GO) analysis describes the function of genes from various aspects, and GO can be divided into three main components, Biological Process (BP), Molecular Function (MF), and Cellular Component. The differential ac4C genes obtained from the analysis were annotated with GOs based on the DAIVD database at the three levels of BP, MF, and CC, respectively, and Fisher's test was used to calculate the significance level (p Value) of each GO to filter out the significant GO Term.

Kyoto Encyclopedia of Genes Genomics (KEGG) analyses are based on gene annotation databases of functional pathways associated with differential genes. The genes of interest in the results of KEGG analyses are those that are related to the actual signaling pathways, and there are direct interactions between the genes. Compared to GO analysis, KEGG analysis is more direct and allows researchers to study the target gene. Compared to GO analysis, KEGG analysis is more direct and allows researchers to study the target gene. The signalling pathway analysis aimed to find signaling pathways significantly enriched for differential ac4C genes based on the KEGG database. The screened differential ac4C genes were subjected to Pathway annotation based on the KEGG database. Fisher's test was used to calculate the significance level (p Value) of the Pathway to screen for significant Pathway Terms for differential ac4C gene enrichment.

### Association analysis and dual-validation

The amount of change log2 (fold change) for all significant differentially ac4C-modified genes was plotted as a horizontal coordinate, and the amount of change log2 (fold change) for all significant differentially expressed genes was plotted as a vertical coordinate for association analysis. Thus, there are four types of association analysis results: Hyperacetylated-down (hyperacetylated-RNA level downregulation); Hyperacetylated-up (hyperacetylated-RNA level upregulation); Hypoacetylated-down (deacetylated-RNA level downregulation); Hypoacetylated-up (Hypoacetylated-RNA levels upregulated).

## Experimental validation

### CCK-8 assay

MNNG/HOS cells were inoculated in 96-well plates at a density of 6000 cells per well, and a gradient concentration of Remodelin intervention was performed after 24 h. After 24 h of intervention, 1–2 h before the test, the medium was replaced with medium that also had 10% CCK-8 (Beyotime, China), and the absorbance at 450 nm was detected after 1–2 h of incubation.

### qRT-PCR analysis

MNNG/HOS cells were intervened with 100 μM Remodelin for 24 h. Intracellular RNA was then extracted using the RNA-Quick Purification Kit (ES science, China), and RNA was reverse transcribed into cDNA using the HiScript® III All-in-one RT Super Mix (Vazyme, China) Reverse Transcription Kit. The qPCR reaction was performed with ChamQ Universal SYBR qPCR Master Mix kit, and the expression levels were detected using a CFX96 Real-Time PCR instrument (Bio-Rad, America). The relative expression of target genes was calculated using the 2^−ΔΔCT^ method. Primer sequences are shown in the Supplementary Material (Supplementary Table S1).

### Statistical analysis

The experimental data were presented as mean ± standard deviation. Software SPSS16.0 and GraphPad Prism 8, Mann–Whitney test and method One-way ANOVA were used to analyze the result of data. A level of P < 0.05 was deemed significant.

## Results

### Flowchart

**Figure 1:**
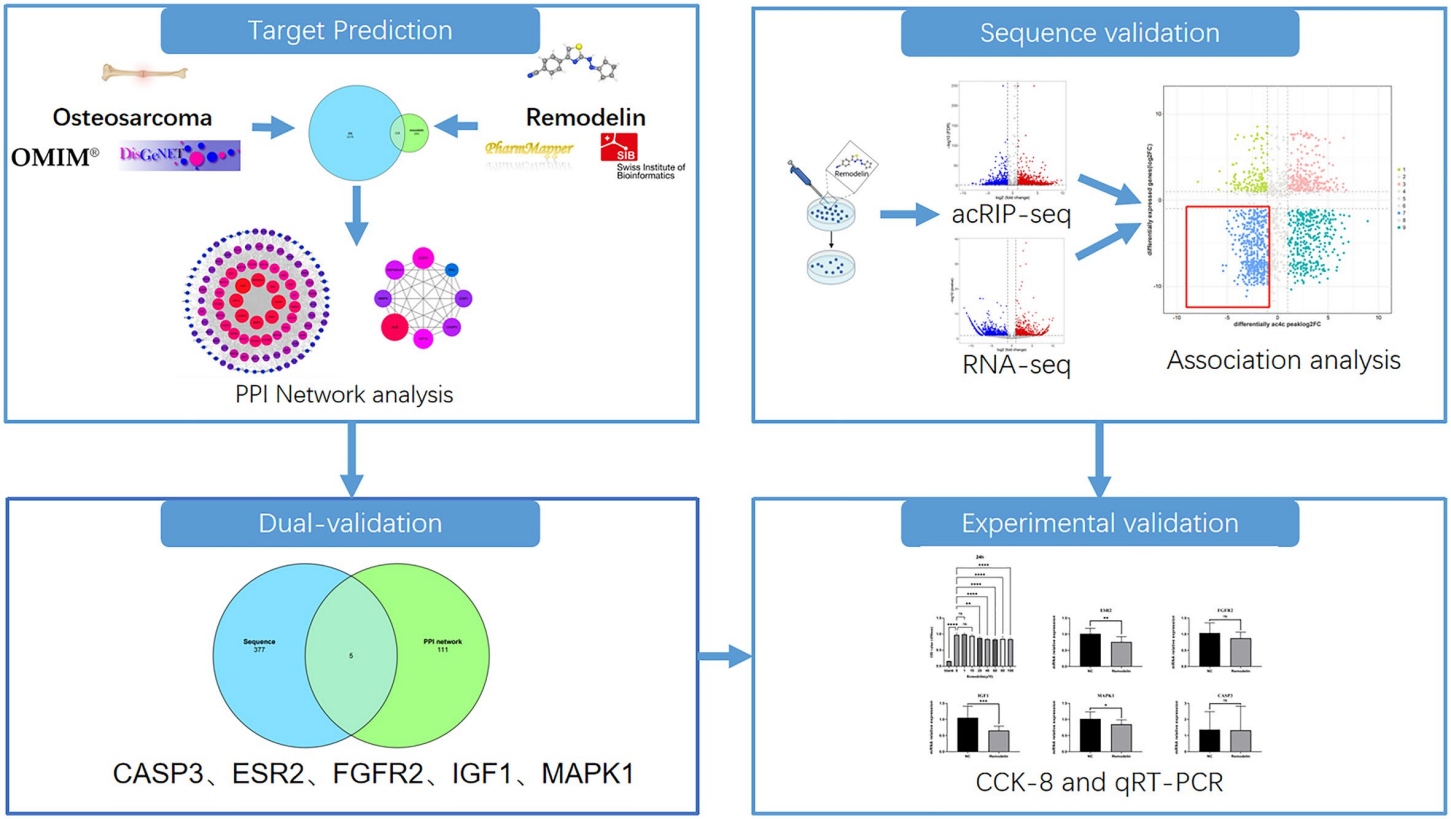
The study workflow.

The research process is shown in Fig. 1.

### Identification of the Remodelin targets

**Figure 2:**
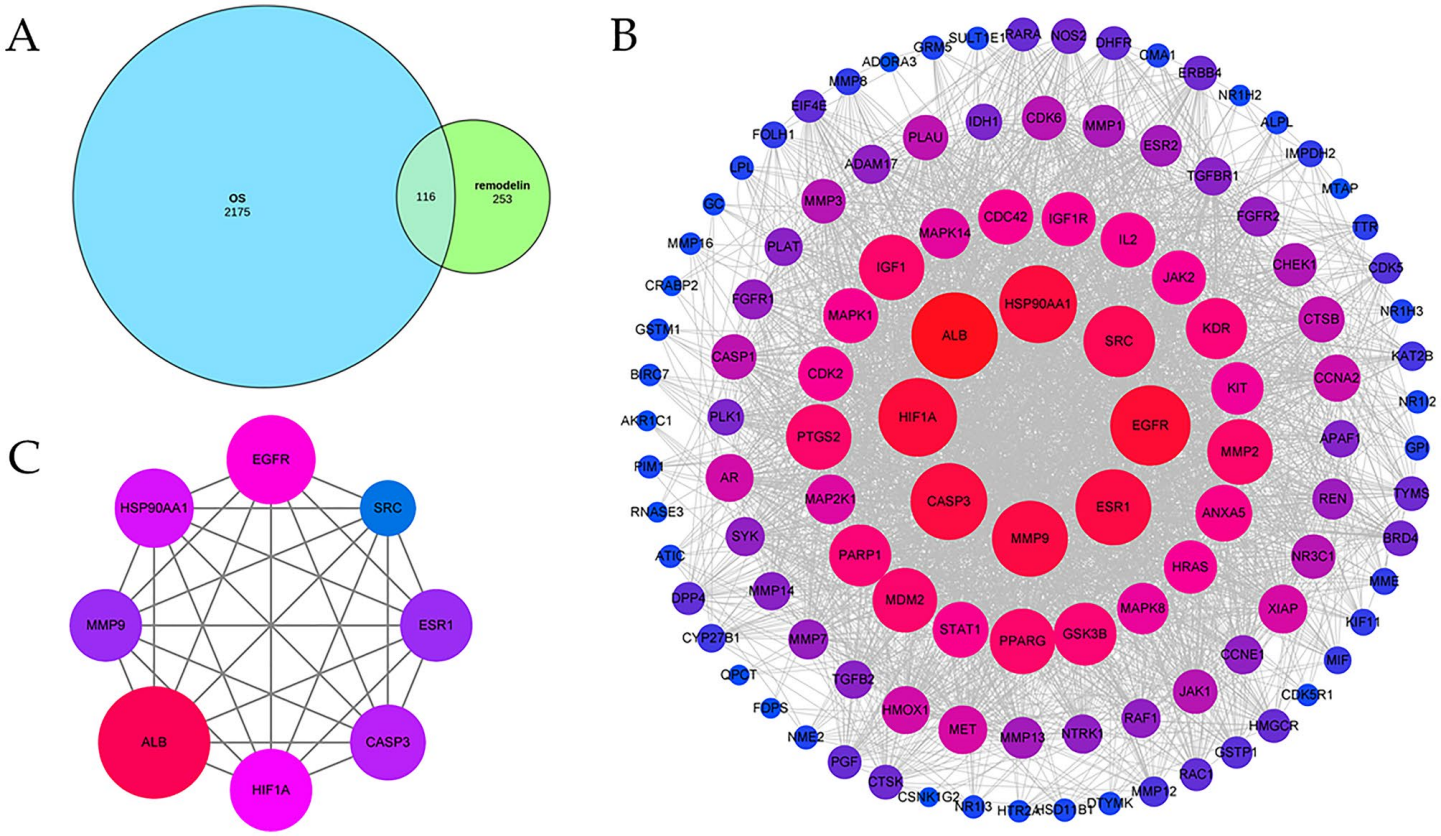
Network pharmacological analysis of Remodelin's targets for OS. (**A**) Venn diagram analyzing overlapping target genes of Remodelin and OS. (**B**) The PPI network is constructed by Cytoscape. The node size and color stand for the size of the degree. Node size is proportional to its degree. The node color is from blue to red and the corresponding degree is gradually larger. (**C**) The top 8 hub genes cluster generated from (**B**).

Remodelin targets were retrieved from 290 in the Pharmmapper database and 109 in the Swiss Target Prediction database. Thirty duplicate targets were removed after merging, resulting in 369 Remodelin targets (Fig. 2A).

### Identification of the OS targets

OS targets were retrieved in the OMIM database for 9 and in the DisGeNET database for 2284. Three duplicate targets were removed after merging, resulting in 2291 OS targets. The two sets of targets obtained above were overlapped and a total of 116 overlapping target genes were found (Fig. 2A).

### Construction of PPI network

The 116 overlapping target genes were imported into the String database to obtain the PPI network, Homo sapiens was selected for the Organism, several evidences were selected for the Meaning of the network, and the Minimum required interaction score was set to medium confidence (0.400). The results (tsv format) were imported into Cytoscape for visualization and analysis of complex relationships between genes. Finally, the top eight targets with the largest degree values were identified as hub genes, which were analyzed individually and set into a network. Out of 116 targets, 114 targets were connected in the network and 2 targets were not connected in the network and the following results were obtained: number of edges: 1379, average node degree: 23.8, avg. local clustering coefficient: 0.604, expected number of edges: 618, PPI enrichment p value: < 1.0e − 16 (Fig. 2B). Eight targets ALB, EGFR, HIF1A, HSP90AA1, CASP3, ESR1, MMP9, SRC were identified as hub genes (Fig. 2C and Table 1).

**Table 1:** Hub genes table for Remodelin on OS by Degree method.

Gene Name	Degree	Topological Coefficient
ALB	77	0.269509252
EGFR	70	0.29380531
HIF1A	68	0.301665799
HSP90AA1	67	0.29184435
CASP3	66	0.309600429
ESR1	65	0.296528251
MMP9	65	0.313236313
SRC	60	0.321559633

### Screening of differential genes for ac4C acetylation modifications caused by Remodelin and analysis of their biological functions

**Figure 3:**
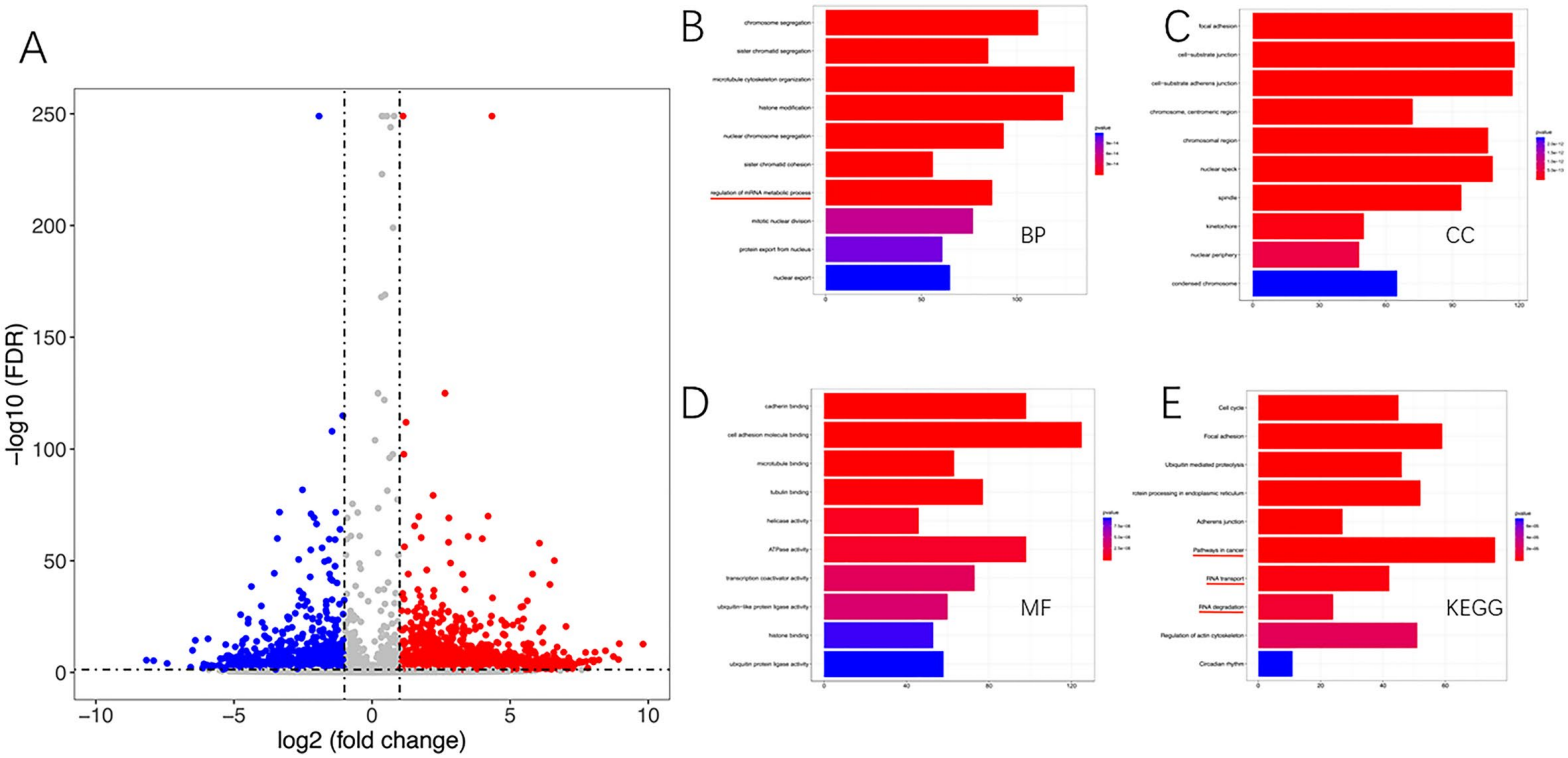
Screening of differential genes for Ac4C acetylation modification and analysis of their biological functions. (**A**) Volcano plots show statistically significant differences ac4C peaks (∣fold change∣ > 2, P < 0.05). Blue dots indicate significantly Hypoacetylated RNAs and red dots indicate significantly hyperacetylated RNAs. (**B**) The BP component of the GO analysis of differentially modified genes, with the colour representing the p value and the bar representing the number of genes corresponding to that go term. (**C**) The CC component of the GO analysis of differentially modified genes, with colour representing the p value and bar representing the number of genes corresponding to that go term. (**D**) The MF component of the GO analysis of differentially modified genes, with colour representing the p value and bar representing the number of genes corresponding to that go term. (**E**) KEGG analysis of differentially modified genes, colour represents p value and the bar represents the signalling pathway corresponding to that KEGG term.

We intervened with U2OS OS cells using Remodelin and another group of cells using DMSO, the total RNA therein was then extracted using Trizol, the RNA was fragmented, and RNA fragments enriched for ac4C acetylation modifications were immunoprecipitated using the ac4C antibody. This portion of RNA was deep sequenced and ac4C peaks were identified using the ExomePeak R package (v2.13.2). The differential ac4C peaks with ∣fold change∣ > 2.0 and p value < 0.05 were selected. A total of 13,736 ac4C-modified peaks with statistically significant and greater than two-fold differences were detected, of which 6938 were Hypoacetylated modifications and 6798 were hyperacetylated modifications. Remodelin acts as an inhibitor of the acetyltransferase NAT10, but its regulatory mechanism may not be as simple as that, and may also lead to hyperacetylation modifications of genes through direct or indirect actions (Fig. 3A).

All the differentially modified genes were subjected to GO and KEGG analyses to explore their biological functions. The GO analysis based on the DAVID database can be divided into three components: BP (biological process), MF (molecular function), and CC (cellular component). Among them, the BP component showed that the differentially modified genes were mostly related to the biological processes of chromosomes: Chromosome segregation, Sister chromatid segregation, Histone modification, Nuclear, chromosome segregation, Sister chromatid cohesion, Regulation of mRNA metabolic process, Mitotic nuclear division, which were also indicated for the regulation of mRNA metabolism process (Fig. 3B), which further proved the regulatory role of Remodelin on mRNA metabolism. The CC component showed that the differentially modified genes were mostly related to Focal adhesion, Cell-substrate junction, Cell-substrate adherens junction, Chromosome, centromeric region, Chromosomal region, Nuclear speck, Spindle (Fig. 3C). The MF component showed that the differentially modified genes were mostly related to Cadherin binding, Cell adhesion molecule binding, Microtubule binding, Tubulin binding, Helicase activity, ATPase activity, Transcription coactivator activity (Fig. 3D). In addition, KEGG analyses also showed that genes differentially ac4C-modified were suggestive of cancer pathways, RNA transport, and RNA degradation, This reinforces the role of Remodelin in the regulation of mRNA metabolism and its potential impact on tumors (Fig. 3E), The results of the bioconfidence analysis provide an indication of the mechanism of action of Remodelin on OS cells.

### Motif analysis and single base site analysis of differential ac4C modifier genes

**Figure 4:**
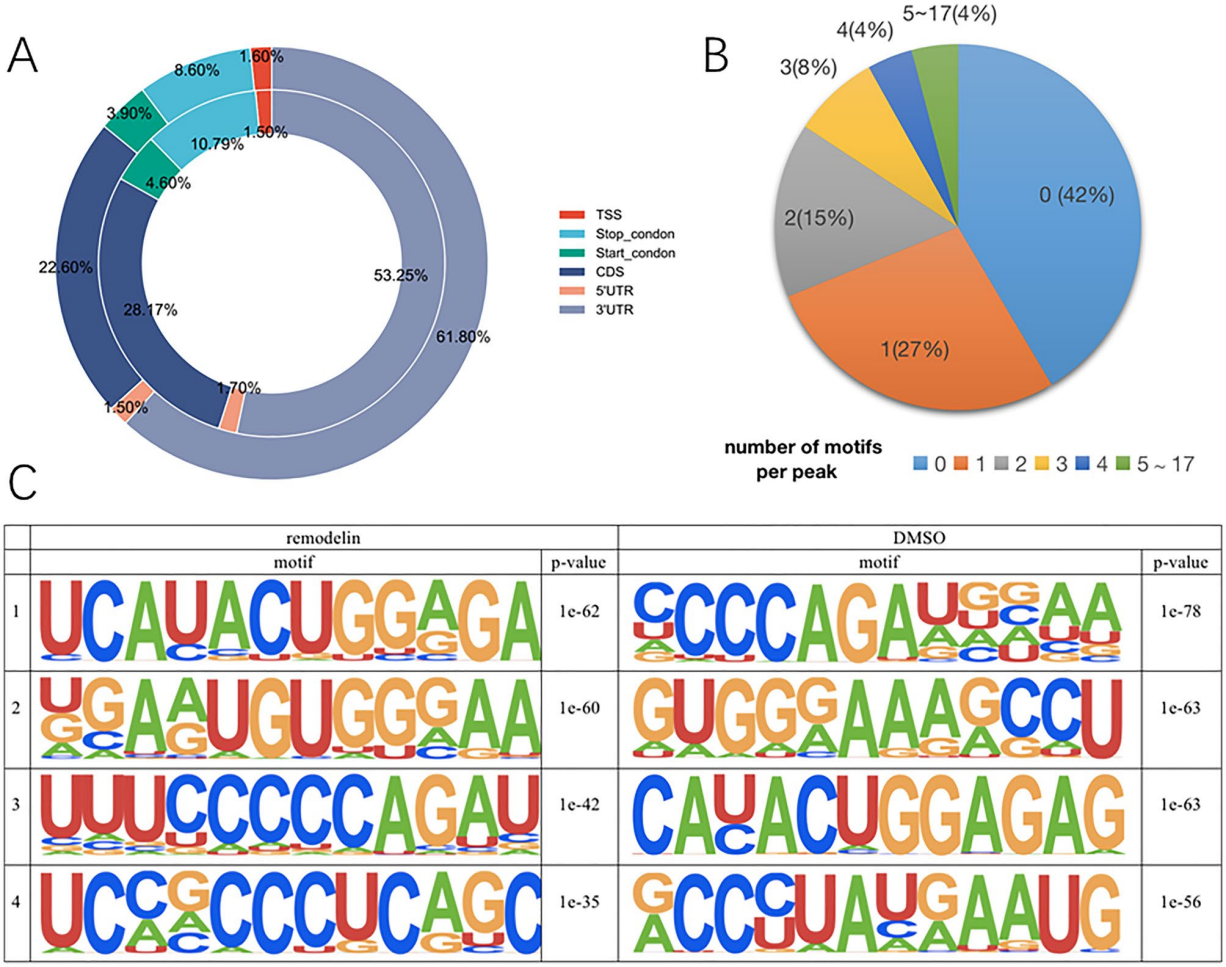
Motif analysis and single base site analysis. (**A**) Pie charts showing the distribution of ac4C Peak across RNA structures in different genetic backgrounds in the outer Remodelin group and the inner DMSO group (Remodelin group vs. DMSO group). The mean values of the percentage of ac4C peaks in the 5ʹUTR, Start Condon, CDS, Stop Condon, and 3'UTR regions of the RNA structure are shown. (**B**) Distribution of peaks with different numbers of motif "CXXCXXCXX" in ac4C modifications among all peaks. (**C**) The first five of the motifs corresponding to the ac4C peaks in the Remodelin and DMSO groups are shown in the table.

We analyzed the distribution of ac4C peaks on RNA structure and found that ac 4C peaks in Remodelin and DMSO groups were mainly enriched in the 3ʹ-untranslated region (3ʹUTR), coding sequence (CDS), and stop codon (StopC), with a slight but insignificant difference between the two groups. This result is slightly different from the results of Arango's experiments, which were mainly distributed in the CDS region 3 (Fig. 4A).

The principle of single base site analysis is to find the position of "CXXCXXCXX" sequences in the peak region, and the number of "CXXCXXCXX" reflects the exact position and number of ac4C modifications in a segment of the peak region, and the number of "CXXCXXCXX" reflects the number of ac4C modifications in a segment of the peak region. The results are an important reference for the study of ac4C modification. Single-base site analysis showed that 58% of the differential ac4C-modified peaks produced after Remodelin intervention had the motif "CXXCXXCXX", and predominantly 1 or 2 motifs were present in the peaks (Fig. 4B). This suggests that the ac4C modification is indeed present in the motif "CXXCXXCXX". ac4C modification is typically found in the motif "CXXCXXCXX"3. The results of motif analysis showed that cytidine was present in large quantities in the motif, and the sequences detected had a high degree of match with "CXXCXXCXX", providing further evidence for ac4C modification of RNA in OS (Fig. 4C).

### Genetic analysis of differential ac4C modifier genes

Analysis of acetylation genes in OS cells of the DMSO group can be approximated as the acetylation modification profile of osteosarcoma cells. We found that the number of exons spanned by each RNA acetylation site, or the number of exons contained in the transcript corresponding to each detected ac4C peak, was predominantly 1 or 2 in terms of exons (Supplementary Fig. S1). After Remodelin intervention was given to OS cells, the number of exons contained in the transcript corresponding to each detected ac4C peak was predominantly 1 or 2, suggesting that each differential ac4C acetylation modification was mostly present in only 1 or 2 RNAs, with good specificity (Supplementary Fig. S2).

At the chromosomal level, ac4C acetylation is modified in all chromosomes, mainly in chromosomes 1, 2, and 3. In contrast, after Remodelin intervention was given to OS cells, there was a significant decrease in the number of ac4C peaks in chromosome 2 compared to the DMSO group (Supplementary Fig. S3). Differential ac4C modification peaks were present from all chromosomes, especially chromosomes 1, 2, 3, and 5, and both Hypoacetylated and hyperacetylated differential modifications were present in abundance. Suggests that Remodelin's action is not limited to purely direct inhibition of NAT10, which indirectly leads to Hypoacetylated RNA. Rather, complex regulation may leads to altered levels of transcriptional modifications of Hypoacetylated and Hyperacetylated, thereby affecting OS development (Supplementary Fig. S4).

### Screening and biological function analysis of differentially expressed RNAs caused by Remodelin

**Figure 5:**
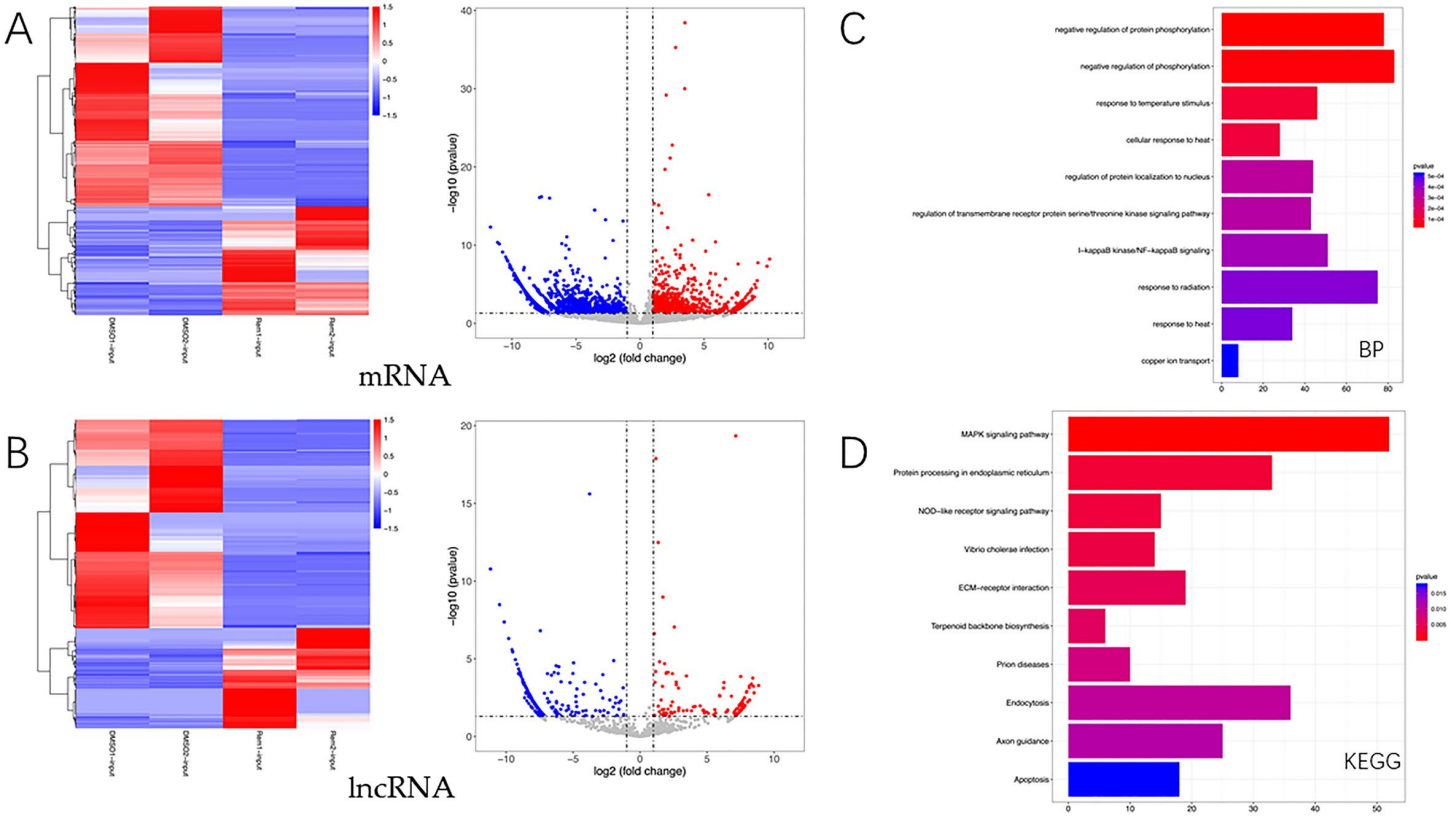
Screening and biological function analysis of differentially expressed RNAs due to Remodelin. (**A**) Volcano and heat maps of differentially expressed genes for mRNA. (**B**) Volcano and heat maps of differentially expressed genes of lncRNA. Red represents relatively high gene expression and blue represents relatively low gene expression. Genes clustered in the same cluster may have similar biological functions. (**C**) BP component in GO analysis of differentially expressed genes of mRNA. (**D**) KEGG analysis of differentially expressed genes of mRNA.

We performed INPUT RNA-seq of RNA from OS cells subjected to Remodelin intervention to obtain sequencing of long-stranded RNA. The differential expression RNA with ∣fold change∣ > 2.0 and p value < 0.05 were selected. A total of 2350 statistically significant mRNAs with greater than two-fold differences were detected, of which 830 were up-regulated and 1520 were down-regulated; A total of 363 statistically significant and greater than two-fold differences in lncRNAs were detected, of which 118 were up-regulated and 245 were down-regulated. The results were analyzed by visualization (Fig. 5A,B). We then performed GO and KEGG analyses on the differentially expressed genes, and GO analyses revealed that in the BP fraction, the differentially expressed genes were mainly associated with the following biological processes: negative regulation of protein phosphorylation, negative regulation of phosphorylation, response to temperature stimulus, cellular response to heat (Fig. 5C). KEGG analysis revealed that differentially expressed genes were mainly associated with the following pathways: MAPK signaling pathway, Protein processing in endoplasmic reticulum, NOD-like receptor signaling pathway, Vibrio cholerae infection, ECM-receptor interaction (Fig. 5D).

### Hypoacetylated-down genes were obtained by association analysis and dual-validation

**Figure 6:**
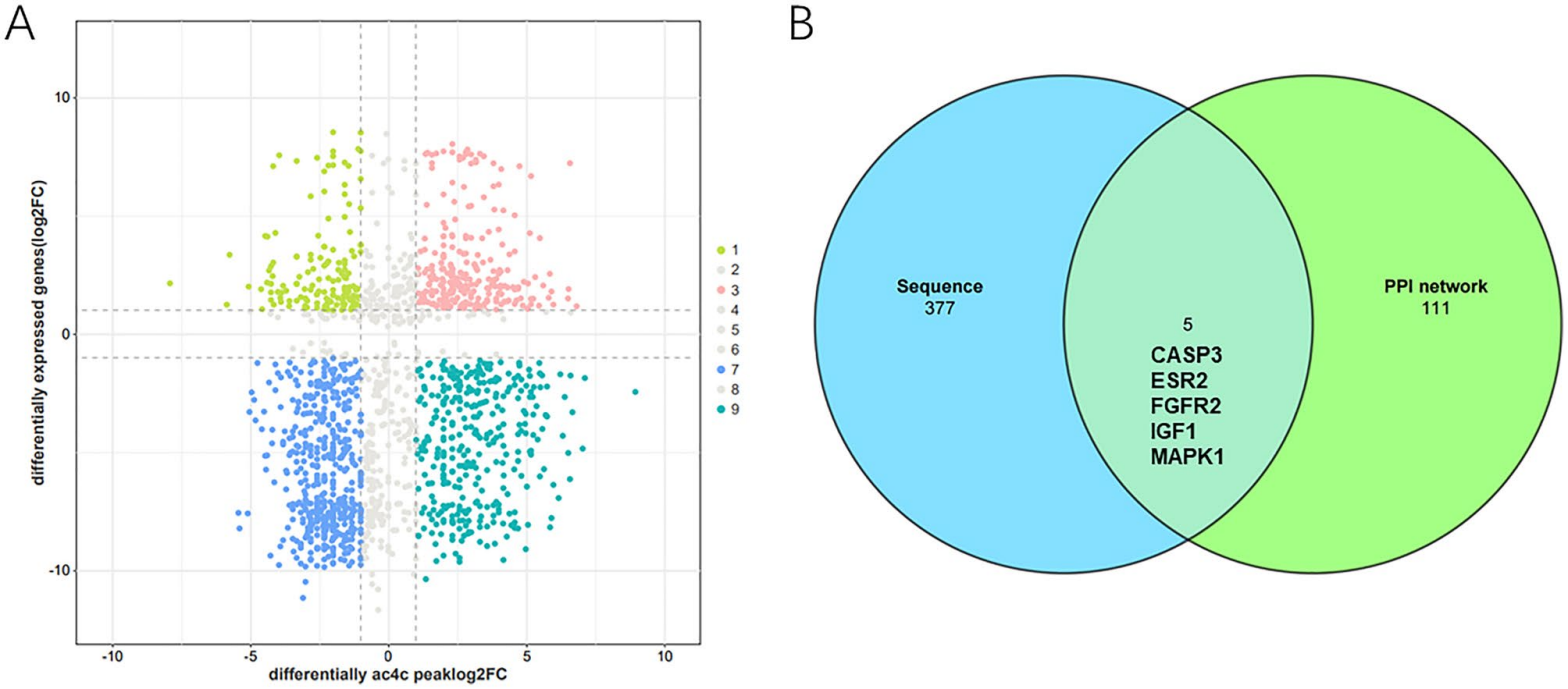
Hypoacetylated-down genes were obtained by Association analysis and Dual-validation. (**A**) Four-quadrant plot of association analysis of differential mRNA modifications with differential gene expression, with vertical coordinates representing the log2 (fold change) of the differential ac4C modification peak and horizontal coordinates representing the log2 (fold change) of the differentially expressed genes. (**B**) Venn plot of PPI network target genes versus Hypoacetylated-down genes.

The amount of change log2 (fold change) in genes with statistically different ac4C modifications was used as the horizontal coordinate, and the amount of change log2 (fold change) in genes with statistically different expressions was used as the vertical coordinate to plot the association analysis. Thus, there are four types of association analysis results: Hyperacetylated-down (hyperacetylated-RNA level downregulation); Hyperacetylated-up (hyperacetylated-RNA level upregulation); Hypoacetylated-down (deacetylated-RNA level downregulation); Hypoacetylated-up (Hypoacetylated-RNA levels upregulated). The gene spots that make ∣fold change∣ ≤ 2.0 turn grey. The top ten mRNAs with the greatest degree of differential ac4C modification change were Hyperacetylated-down 6 genes, respectively: ZHX1, CTDSP1, UGP2, HSPB11, MTFR1L, MTUS1; Hyperacetylated-up 3 genes: FXR1, MYC, DNAAF3; and 1 Hypoacetylated-down gene: IFNGR1 (Fig. 6A). A total of 382 Hypoacetylated-down genes were identified. As Remodelin is an inhibitor of NAT10, it inhibits the acetyltransferase activity of NAT10, leading to Hypoacetylated mRNA. According to the literature 3, Deacetylation can lead to a decrease in mRNA translation efficiency and a decrease in stability, so it is highly likely that mRNAs in the Hypoacetylated-down type are potential targets for Remodelin. Our previous study also confirmed FNTB as a Remodelin target gene in the Hypoacetylated-down type12. The 116 target genes of the PPI network intersected with 5 of the 382 Hypoacetylated-down genes in the sequencing results, suggesting that these 5 target genes (Fig. 6B), CASP3, ESR2, FGFR2, IGF1, and MAPK1, do indeed have a state of hypoacetylation and low expression. Inhibition of NAT10 by Remodelin may lead to hypoacetylation of these five genes and thus to low expression. Dual validation by network pharmacology and sequencing suggest that Remodelin is likely to exert its therapeutic effects through these five genes.

### Remodelin inhibits the proliferation of OS cells

**Figure 7:**
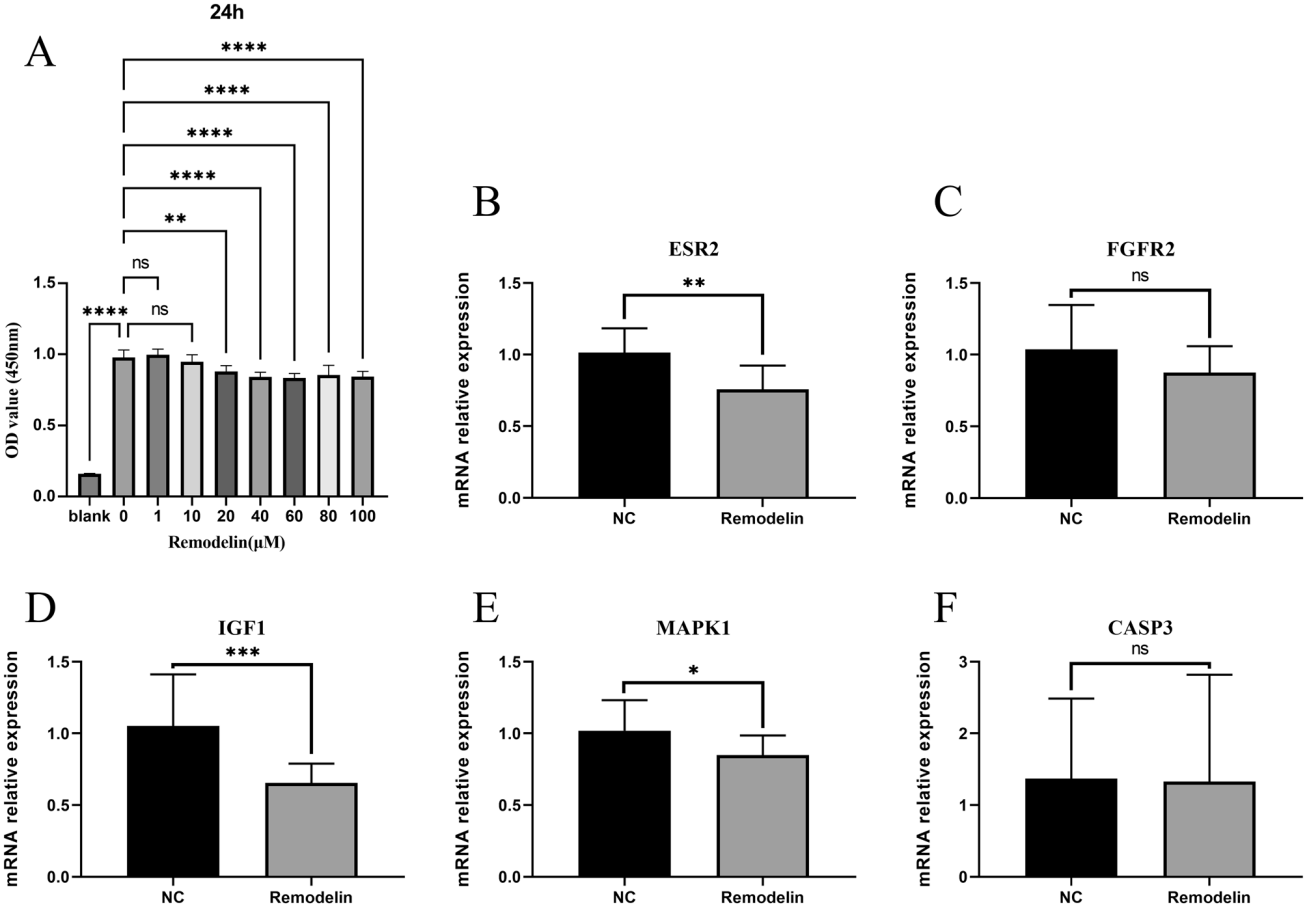
Remodelin inhibits the proliferation of OS cells by suppressing the expression of ESR2, IGF1 and MAPK1. (**A**) CCK-8 assay for the determination of Remodelin toxicity to OS cells. (**B-F**) The mRNA relative expression of ESR2, FGFR2, IGF1, MAPK1, CASP3. The data are presented as the mean ± SD of six independent experiments.*P < 0.05, **P < 0.01, ***P < 0.001.

We intervened OS cells with graded concentrations of Remodelin (1, 10, 20, 40, 60, 80, 100 μM) for 24 h. The cytotoxicity was detected by CCK-8, and the results showed that Remodelin could inhibit OS cells from 20 μM onwards in a concentration-dependent manner compared with the control group (0 μM) (Fig. 7A). We then selected a concentration of 100 μM for the target validation of Remodelin.

### ***Remodelin reduces mRNA expression of ESR2, IGF1, MAPK1***

We used qRT-PCR to validate the five key target genes screened and found that Remodelin indeed significantly reduced the expression of ESR2, IGF1, and MAPK1. In addition, FGFR2 expression was reduced but not significantly different, and CASP3 expression remained unchanged (Fig. 7B–F). ESR2, IGF1 and MAPK1 all belong to the MAPK signaling pathway, which shows that remodulin is likely to have a therapeutic effect on OS through the MAPK signaling pathway.

## Discussion

Since its inception in the 1980s, the conventional approach to treating OS, which includes surgical intervention and chemotherapy, has proven to be highly effective in ensuring long-term survival for over 60% of patients with localized disease, but for patients with relapsed and metastatic OS, the original treatment method cannot produce effective therapeutic effects^13^. Chemotherapy has made significant progress as a conventional treatment for cancer. However, the effectiveness of chemotherapy still faces serious clinical challenges due to the lack of targets and limited bioavailability, as well as the susceptibility to drug resistance^14^. In the area of targeted therapies, Therapeutic targeting of several prevalent genetic changes observed in OS, including TP53 break-down translocations and RB1 deletions that disrupt the expression of crucial tumor-suppressor proteins, has posed significant challenges. Epigenetics has proposed therapies that target key genes through a genetic approach, as well as treatments that influence gene expression through genetic modifications, thus opening up new possibilities for diagnosing and treating diseases. Ac4C is an emerging form of epitranscriptome that has been reported to contribute to the correct reading of codons during translation and improve translation efficiency and mRNA stability^3,15^. Extensive documentation exists regarding the correlation between Ac4C acetylation modifications and tumors^2,5,8,9^. No research has been conducted on OS and other related conditions. Liebich et al.^16^ conducted a study on cancer biomarkers discovered a notable rise in the nucleoside count in the urine of cancer patients, particularly in modified nucleosides (such as ac4C), indicating the efficacy of modified nucleosides (e.g., ac4C) as reliable markers for tumor diagnosis.

NAT10, the unique ac4C mRNA-modified acetyl transferase identified, contains 1025 amino acids with a molecular weight of 116 kD. NAT10 catalyzes mRNA acetylation in coding sequences (CDS) to improve mRNA stability and translation efficiency^3^. NAT10 has been reported to be associated with multiple cancers such as epithelial ovarian cancer^17^, Hepatocellular carcinoma^18–21^, breast cancer^22^, colorectal cancer^23,24^, and acute myeloid leukaemia^25^. However, most of these studies describe other functions of non-mRNA acetyltransferases. The role of NAT10 in OS and the specific association between mRNA ac4C modifications and OS pathogenesis remain unelucidated.

It has been documented that Remodelin has the ability to improve the cellular defects associated with HutchinsoneGilford progeria syndrome (HGPS) by inhibiting NAT10 and restoring the nuclear structure of patient cells derived from HGPS through microtubule reorganization^11,26^. Remodelin hinders the growth and movement of cells, leading to the cessation or programmed cell death in different types of cancer cells^19,20,22,27^. Remodelin is a putative small molecule inhibitor of the RNA acetyltransferase NAT10, but biophysical analyses by Shrimp et al.^28^ did not find direct evidence of the interaction of remodeling proteins with the NAT10 acetyltransferase active site, suggesting that the chemotype of remodeling proteins may interact with a wide range of protein targets in the cell, and suggesting that remodeling proteins should not be used as specific chemical inhibitors of NAT10-catalysed RNA acetylation. So our team, based on the discovery of Remodelin's inhibitory effect on OS, continued to explore Remodelin's other targets besides NAT10 in the hope of gaining a comprehensive understanding of the mechanism of Remodelin's action in OS. Our study identified five genes identified as key therapeutic targets of Remodelin for OS: ESR2 (Estrogen receptor beta), FGFR2 (Fibroblast growth factor receptor 2), IGF1 (Insulin-like growth factor I), MAPK1 (Mitogen-activated protein kinase 1), CASP3 (Caspase-3). Vitro experiments demonstrated that Remodelin significantly reduced the expression of ESR2, IGF1, and MAPK1 in OS cells. Among them, IGF1 and MAPK1 belong to the MAPK signaling pathway, so it is evident that remodulin is likely to have a therapeutic effect on OS through the MAPK signaling pathway. In addition, FGFR2 showed a decreasing trend but was not significant, and CASP3 expression did not seem to be affected by Remodelin.

MAPK1 is a serine/threonine kinase that is an important component of the MAP kinase signaling pathway. MAPK1/ERK2 and MAPK3/ERK1 are two MAPKs that play an important role in the MAPK/ERK cascade. The MAPK/ERK cascade regulates transcription, translation, and cytoskeletal rearrangement according to the cellular environment. Over ten years ago, scientists discovered that the MAPK signaling pathway is not functioning properly in OS^29^. On the therapeutic side, many drugs can produce therapeutic effects on OS through the MAPK signaling pathway, known as Macrophage migration inhibitory factor^21^, Escin^30^, G721-0282^31^.

Insulin-like growth factor (IGF), which is structurally and functionally similar to insulin but has higher growth-promoting activity. IGF is produced by osteoblasts and acts through its receptor IGF1R to activate downstream protein kinases, including those in the MAPK/ERK 1/2 signaling pathway that regulate cancer cell growth and survival^32^. IGF 1 R has also been reported as a potential target for the treatment of high-grade OS^33^. Furthermore, IGF 1 R inhibition enhances the response of certain OS cell lines to adriamycin chemotherapy^34^. Thus Remodelin reduces the expression of IGF1 thereby attenuating the activation of IGF1R and thus inhibiting the development of OS.

Loss of estrogen or defective estrogen receptor (ER) function inhibits osteoblast growth and impairs osteogenesis. Because of the important role of estrogen receptors in bone formation, whether controlling estrogen receptors regulates new bone formation and affects the prognosis or sensitivity to chemotherapy in bone tumors is a question that deserves further study^35^. Our study found that Remodelin reduces the expression of ESR2, thus it may be able to inhibit the formation of new bone in OS patients, which in turn inhibits tumor growth.

Although our experimental results showed that Remodelin did not significantly reduce FGFR2 expression. Tyrosine protein kinase is a cell surface receptor of fibroblast growth factors (FGF) and plays an important role in regulating cell proliferation, differentiation, migration and apoptosis, as well as embryonic development. Heterozygous loss of FGFR2, one of the Tyrosine-protein kinases, was observed in high-grade OS at 10q26^36^. Genetic variation of FGFR2, which plays an important role in bone morphogenesis, has been shown to underlie skeletal dysplasias^37^.

There is a strong correlation between these targets, which we obtained by means of network pharmacology and acRIP-seq. IGF1 may be involved in the MAPK signaling pathway as a ligand binding to the IGF1R receptor, while FGFR may be involved in the MAPK signaling pathway as a tyrosine kinase receptor, suggesting that the MAPK signaling pathway may be a mechanism for Remodelin function. These results reveal the potential therapeutic target of remdesivir in treating osteoporosis and provide a theoretical basis for remdesivir to treat OS.

We analyzed the distribution of ac4C peaks on RNA structure and found that ac 4C peaks in Remodelin and DMSO groups were mainly enriched in the 3ʹ-untranslated region (3'UTR), coding sequence (CDS), and stop codon (StopC), with a slight but insignificant difference between the two groups. This result is slightly different from the results of Arango’s experiments, which were mainly distributed in the CDS region^3^(Fig. 4A). In 2018, Arango et al.^3^ showed that ac4C was present in more than 4000 regions of the human transcriptome. In human HeLa cells, ac4C is predominantly enriched in the coding sequence (CDS) region. However, since only Arango et al. have published research results on human mRNA ac4C, the credibility of this experiment and its results deserve further verification^38^.

However, our study has some limitations: first, our study only screened and validated Remodelin's targets of action in network pharmacology and in vitro experiments, and no vivo experiments were conducted to further validate the results. Secondly, there was no comparison or combination with existing anti-OS drugs, which did not reflect the advantages of Remodelin over other drugs. In future studies, we will further explore its mechanism of action to provide better experimental data for clinical application.

## Conclusion

In summary, we predicted ESR2, FGFR2, IGF1, MAPK1 , and CASP3 as therapeutic targets of Remodelin for OS by network pharmacology and sequencing technology. ESR2, IGF1, and MAPK1 were further validated as key therapeutic targets of Remodlin by vitro experiments. This reveals the target of Remodelin's pharmacological action on OS and provides new ideas for the treatment of OS.

### Supplementary Information

Supplementary Information.

## Data Availability

The original data for this study are available by contacting the corresponding authors.
