# Supplementary material for: Revealing the pharmacological effects of Remodelin against osteosarcoma based on network pharmacology, acRIP-seq and experimental validation
Source: Sci Rep. 2024 Feb 13;14:3577. doi: 10.1038/s41598-024-54197-4 (PMC10861577; doi:10.1038/s41598-024-54197-4)

Supplementary Table S1

| gene | Forward(5'-3') | Reverse(5'-3') |
| --- | --- | --- |
| ESR2 | GCTGAACGCCGTGACCGATG | ACAGGAGCATCAGGAGGTTAGCC |
| FGFR2 | TAGAGCCAGAAGAGCCACCAACC | TCACGGCGGCATCTTTCAACAG |
| IGF1 | TGTCCTCCTCGCATCTCTTCTACC | CCTGTCTCCACACACGAACTGAAG |
| MAPK1 | TCGCCGAAGCACCATTCAAGTTC | TCCTGGCTGGAATCTAGCAGTCTC |
| CASP3 | CATGGAAGCGAATCAATGGACT | CTGTACCAGACCGAGATGTCA |
| beta-actin | CCTGGCACCCAGCACAAT | GGGCCGGACTCGTCATAC |

Supplementary Figure S1


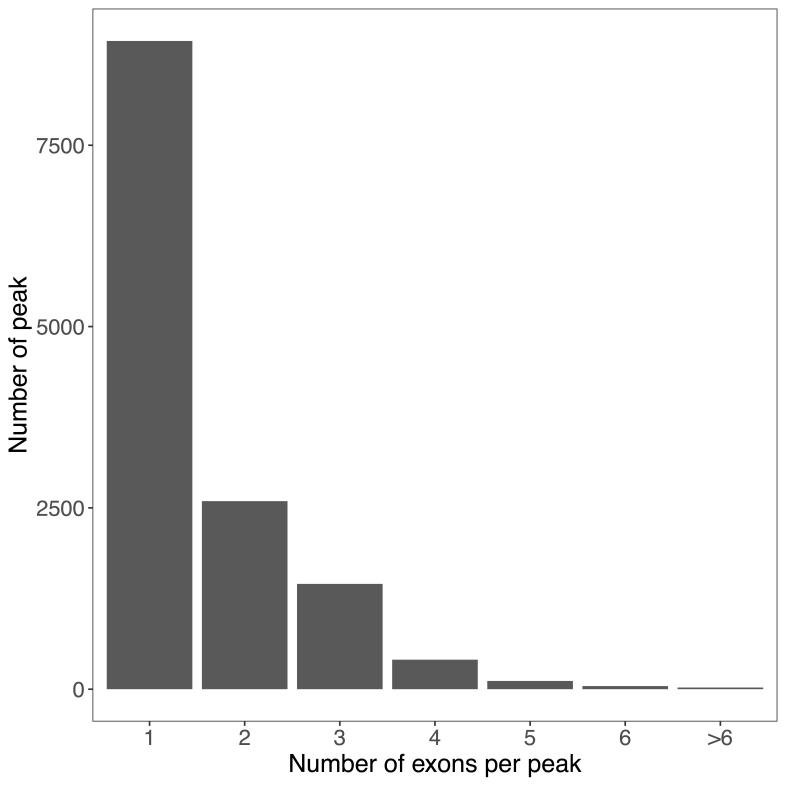


Supplementary Figure S2


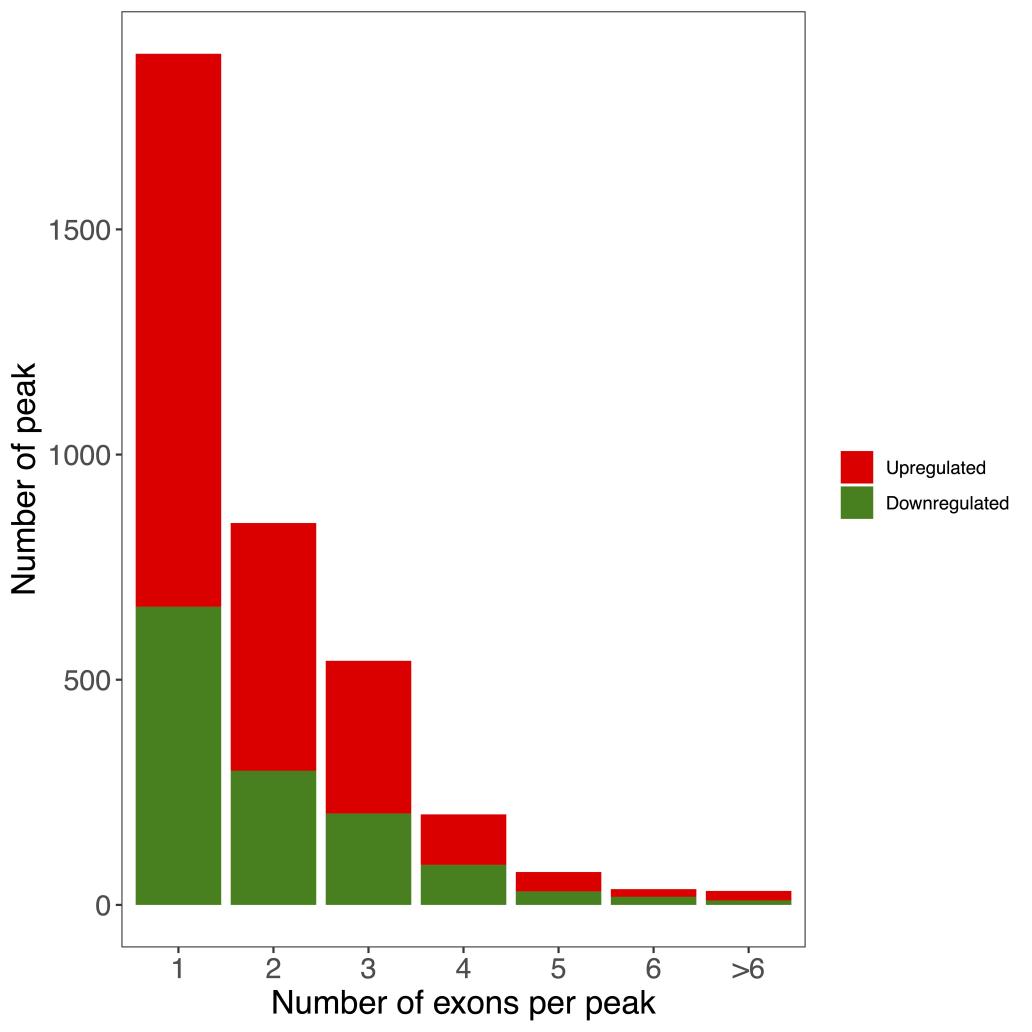


Supplementary Figure S3

A

B


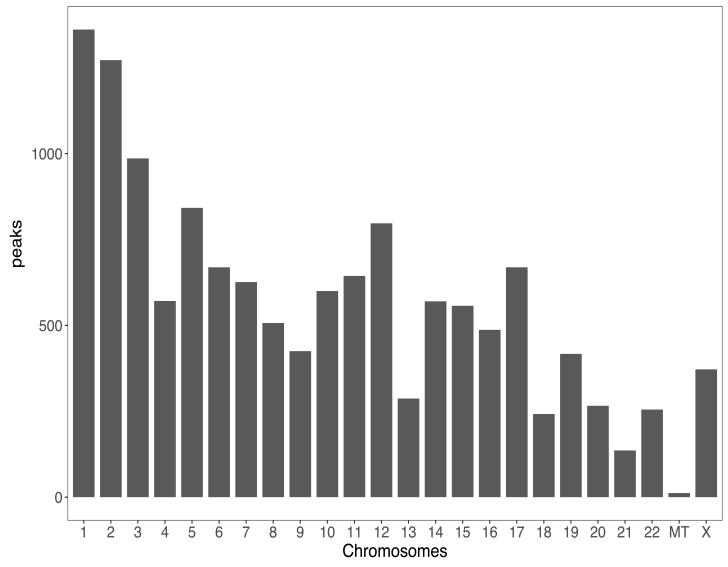

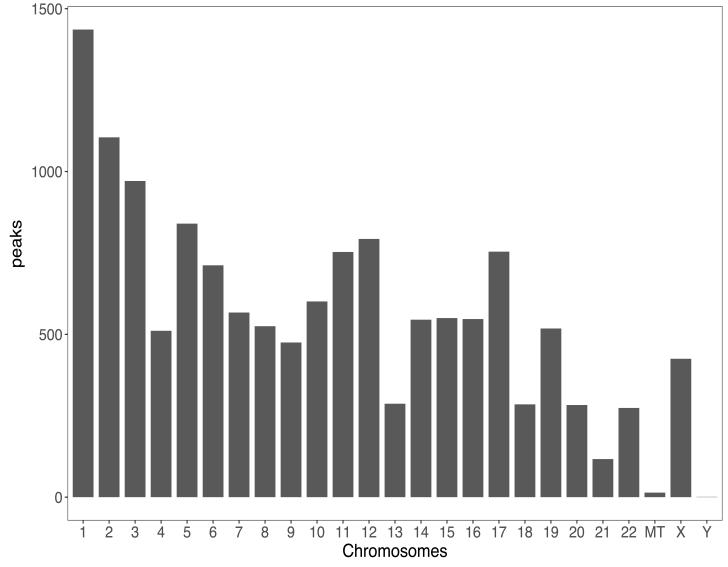


dmso

remodelin

Supplementary Figure S4


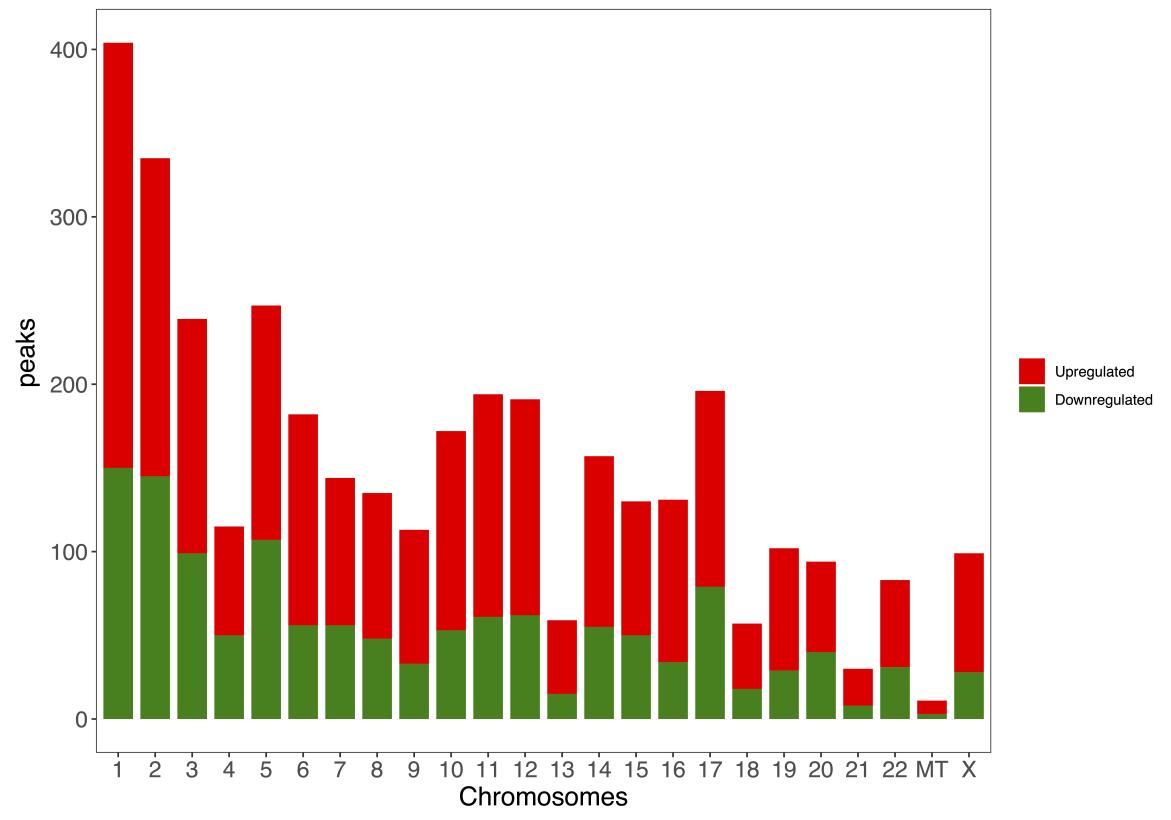

Supplement: Supplementary file 1 — Supplementary Information. [file 41598_2024_54197_MOESM1_ESM.docx]
